# Supplementary material for: Consequences of producing DNA gyrase from a synthetic gyrBA operon in Salmonella enterica serovar Typhimurium
Source: Mol Microbiol. 2021 Feb 27;115(6):1410–29. doi: 10.1111/mmi.14689 (PMC8359277; doi:10.1111/mmi.14689)
Supplement: Supplementary file 1 — Supplementary Material [file MMI-115-1410-s001.pdf]

Consequences of producing DNA gyrase from a synthetic *gyrBA* operon in *Salmonella enterica* serovar Typhimurium

German Pozdeev, Aalap Mogre and Charles J Dorman*

SUPPLEMENTARY FILES

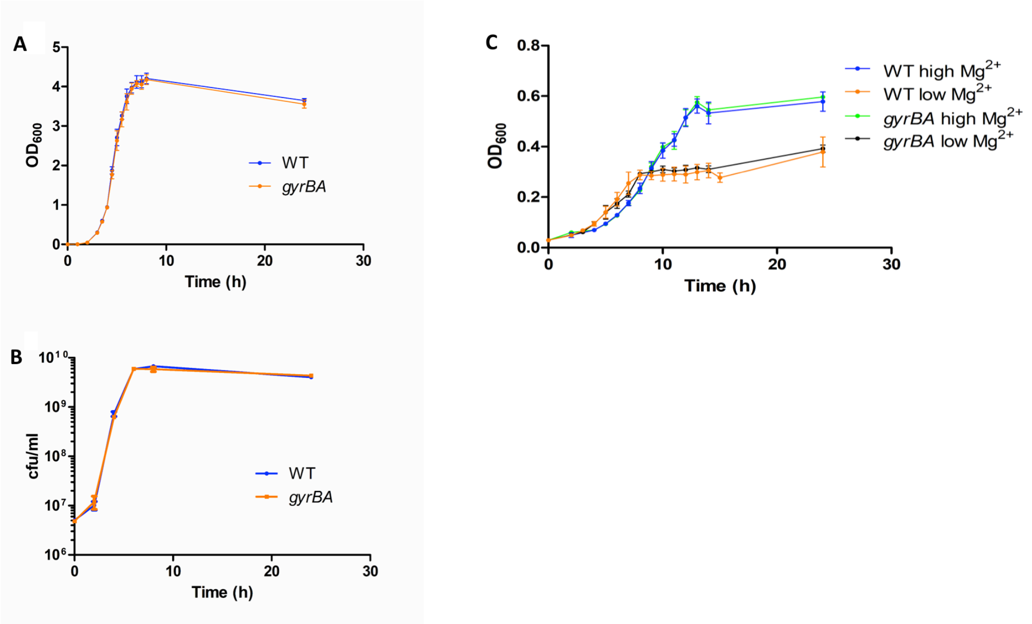

Fig. S1. Growth characteristics of SL1344 *gyrBA*.

A. Growth of the *gyrBA* strain as measured by absorbance at 600nm. OD_{600} measurements were made every hour until 3 h, then every 30 min until 8 h and lastly at 24 h. B. Growth of the *gyrBA* strain as measured by viability counts. Dilutions of bacterial cultures were spread on agar plates, incubated at 37°C and colonies were counted. C. Growth of the WT and the *gyrBA* strains as measured by absorbance at 600 nm in minimal medium N. Pre-conditioned culture was subcultured into 25 ml of fresh minimal medium of the required Mg^{2+} concentration, normalizing to an OD_{600} of 0.03. OD_{600} values were measured every hour from 2 h until 15 h and at 24 h. All plots are the results of at least three biological replicates, error bars represent standard deviation.

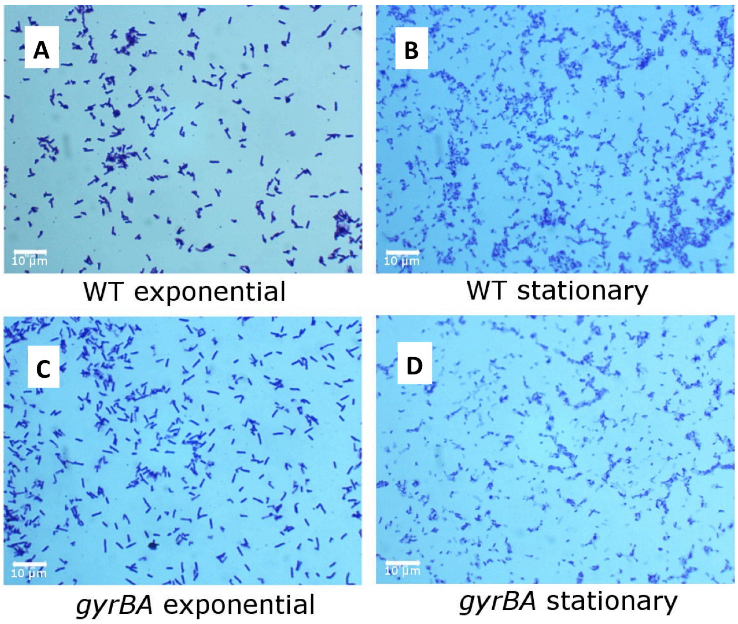

Fig. S2. Cell morphology of SL1344 *gyrBA* at the exponential and stationary phases of growth.

Bacteria were harvested at the mid-exponential or at the late stationary phases of growth, washed with PBS, heat-fixed, stained with crystal violet and viewed under 1000x magnification with an oil immersion lens. Standard rod-shaped *Salmonella* cells were observed in the WT and the *gyrBA* strains. All images are representative of three biological replicates. A 10 µm scale bar is given for reference. Cell morphology in LB liquid cultures is shown for wild type SL1344 in exponential phase (A), stationary phase (B), SL1344 *gyrBA* in exponential phase (C) and stationary phase (D).

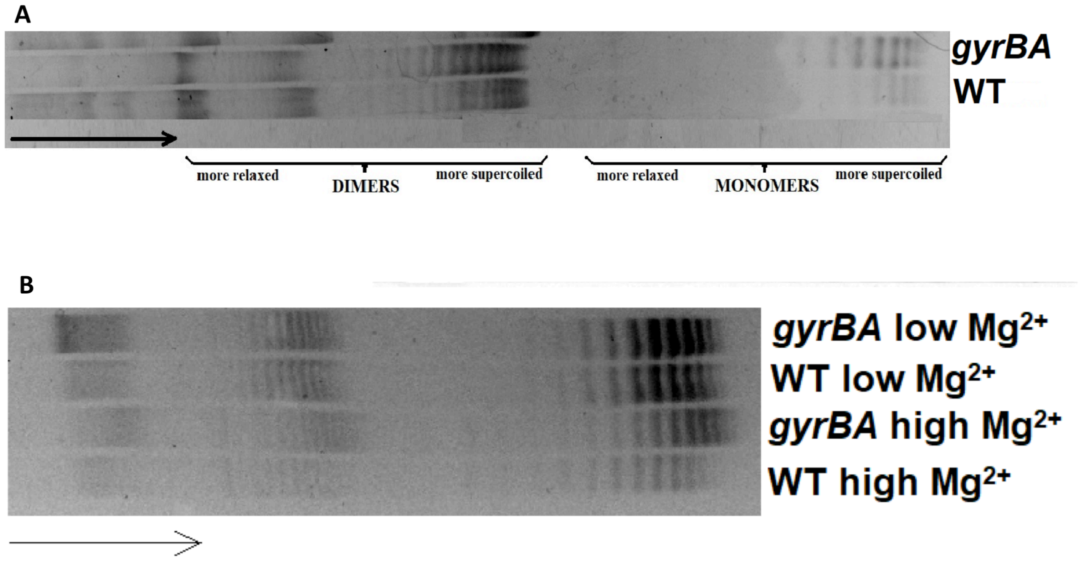

Fig. S3. Full gel data for reporter plasmid DNA supercoiling in SL1344 and SL1344 *gyrBA*.

Samples of pUC18 plasmid were extracted from the WT and the *gyrBA* at the stationary growth phase and run on a 0.8% agarose gel containing 2.5 µg/ml chloroquine to separate pUC18 topoisomers according to the degree of DNA supercoiling. Arrow shows the direction of electrophoretic flow with the more supercoiled plasmid topoisomers at the right of the gel. A) Global DNA supercoiling pattern of the WT and the *gyrBA* when grown in LB. The positions of relaxed and supercoiled plasmid topoisomer dimers and monomers are shown. B) Global DNA supercoiling pattern of the WT and the *gyrBA* when grown in minimal medium N with high (10 mM) Mg^{2+} and low (10 µM) $Mg^{2+.}$ Plasmid topoisomer monomers are at the righthand end of the distribution; dimers and higher order oligomers are to the left.

Table S1 Oligonucleotides used in this study

Name	5'-3' sequence
**Genetic mutation confirmation primers**:	
gyrA_check_Pf2	GACTAAGGTAGCGGTAAATG
gyrA_check_Prev2	GTAGATGACGAAGAACTCG
gyrB_check_Pf2	CAACGAATCCATTCCGATG
gyrB_check_Prev2	CTGATGAGCAGACTGTAAC
gyrA check del R	GCATTGTCTGGCTGCATTC
gyrB check del R	CTTTGTCAGCGCAATTAGC
gyrA_midcheck	CGATGGTGTCATACACTG
gyrB_midcheck	GAACGGTCATGATCACTTC
**qPCR primers**:	
RT_gyrB_F	CTCGTTCAGCTCGGTAATCAG
SL_gyrB_R	ATGATTGGTCGTATGGAGCG
SL_gyrA_qPCR_Pf	CAGCGGTACCGTGAAGAAA
SL_gyrA_qPCR_Prev	AGCATGACTTCGTCAGAACC
RT_hemX_F	CGCCTGACGGTATGTTTCTT
RT_hemX_R	CCCAACCAGGACGTCTATTTAC
**Deletion mutations – *kan* insertions**	
Kan_gyrA_Pf	CCCTCGCACAGCAATAACATTACTCGTCAGCGTCATCCGCCATATGAATATCCTCCTTAG
Kan_gyrA_Prev	CTTTGAATCCGGGATACAGTAGAGGGATAGCGGTTAGATGGTGTAGGCTGGAGCTGCTTC
Kan_gyrB_Pf	GGCCGGGGATTAAGGCAGGTTAAATATCGATATTCGCTGCCATATGAATATCCTCCTTAG
Kan_gyrB_Prev	ACGGATTAACCCAAGATTAAATGAGCGAGAAACGTTGATGGTGTAGGCTGGAGCTGCTTC
gyrA*::*kan* insertion downstream of *gyrB	
Kan ins gyrA F	GGCAAAGAAAAAGGGCCGGATATCCGGCCCTCGCACAGCAGTGCCACCTGCATCGAT
Kan ins gyrA R	AAGCGATGACGACGTTGCGGATGACGCTGACGAGTAATGTCATATGAATATCCTCCTT
gyrB.int.gyrA::kan_Pf	TCATGATGCCCCGGCCAACCAGCGGTAGGCCGGGGATTAAGTGTAGGCTGGAGCTGCTTC
gyrB.int.gyrA::kan_Prev	GGAGAACGCCCTGAAAGCAGCGAATATCGATATTTAACCTAGTAGAGGGATAGCGGTT

For *kan* insertion primers, the black portion is an annealing end and the red portion is an overhanging end.
